# High-Efficient Spin Injection in GaN at Room Temperature Through A Van der Waals Tunnelling Barrier

**DOI:** 10.1186/s11671-022-03712-5

**Published:** 2022-08-15

**Authors:** Di Lin, Wenyu Kang, Qipeng Wu, Anke Song, Xuefeng Wu, Guozhen Liu, Jianfeng Wu, Yaping Wu, Xu Li, Zhiming Wu, Duanjun Cai, Jun Yin, Junyong Kang

**Affiliations:** 1 Department of Physics, College of Physical Science and Technology, Engineering Research Center for Micro-Nano Optoelectronic Materials and Devices at Education Ministry, College of Chemistry and Chemical Engineering, Jiujiang Research Institute, Xiamen University, Xiamen, 361005 People’s Republic of China; 2 Collaborative Innovation Center for Optoelectronic Semiconductors and Efficient Devices, Jiujiang Research Institute, Department of Physics/Pen-Tung Sah Institute of Micro-Nano Science and Technology, Xiamen University, Xiamen, 361005 People’s Republic of China

**Keywords:** Van der Waals tunnelling barrier, Boron nitride, Spin injection, Spintronic devices

## Abstract

Achieving high-efficient spin injection in semiconductors is critical for developing spintronic devices. Although a tunnel spin injector is typically used, the construction of a high-quality tunnel barrier remains a significant challenge due to the large lattice mismatch between oxides and semiconductors. In this work, van der Waals h-BN films with the atomically flat interface were engaged as the tunnel barrier to achieve high spin polarization in GaN, and the spin injection and transport in GaN were investigated systematically. Based on the Hanle precession and magnetic resistance measurements, CoFeB was determined as an optimal spin polarizer, bilayer h-BN tunnelling barrier was proven to yield a much higher spin polarization than the case of monolayer, and appropriate carrier concentration as well as higher crystal equality of n-GaN could effectively reduce the defect-induced spin scattering to improve the spin transport. The systematic understanding and the high efficiency of spin injection in this work may pave the way to the development of physical connotations and the applications of semiconductor spintronics.

**Supplementary Information:**

The online version contains supplementary material available at 10.1186/s11671-022-03712-5.

## Introduction

Semiconductor spintronic devices, which combine electrical charge and spin as information carriers [1], bring new prospects for developing next-generation integrated circuits [2]. III-nitride semiconductors such as GaN, which are widely used in high-performance electronic and optoelectronic devices [3–6], are also promising candidates for energy-efficient spintronic applications. Electrical spin injection is a common route towards functionalized semiconductors spintronic devices [1]. To eliminate the conductivity mismatch between ferromagnetic metals (FM) and semiconductors [7, 8] and achieve high-efficient spin injection and transport, an extremely thin dielectric layer should be inserted as a tunnelling layer at the metal–semiconductor interface [9]. The conventional tunnelling layers used in spintronic devices are oxides, such as Al_2_O_3_ [5, 6, 10] and MgO [11–13], which can improve the tunnelling spin polarization by regulating the height of the tunnelling barriers [14–16]. However, achievement of high spin selectivity is still faced with difficulties including pinholes caused by non-uniform thickness of oxides [17, 18], and spin scattering induced by lattice mismatch [12].

Van der Waals tunnelling is considered an effective strategy to overcome the above problems. Recently, h-BN has been proven to be a promising alternative for the tunnelling layer due to its ultra-wide bandgap, controllable thickness, and high environmental stability [19]. Existing researches have also revealed that h-BN possesses an atomically flat surface free of dangling bond and charged impurity, resulting in consistent insulating property throughout the film [20, 21]. Even atomically thin h-BN can be utilized as an excellent dielectric layer with a high breakdown field [22]. Furthermore, as a two-dimensional layered material, h-BN provides a clean van der Waals interface that can well avoid the lattice mismatch during the subsequent growth of FM [23]. Theoretically, a first-principle study predicted a spin injection efficiency up to 100% in Ni/h-BN/graphene heterostructures [24]. Experimentally, a spin relaxation time of 56 ps ~ 1.86 ns could be achieved at room temperature (RT) [23, 25–29], with the highest spin polarization of 31% in graphene flakes [28]. Although h-BN has shown good performance in the spintronic devices with two-dimensional graphene [19], its application to other systems, especially to the three-dimensional semiconductors for spin injection, is devoid. Simultaneously, both h-BN and GaN are nitride materials, and some groups have well performed the epitaxy of h-BN on GaN [30, 31], which indicates a possibility for the in situ integrated growth of the whole spin injection structure including semiconductor and spin injector. This will shed more light on the development of spintronic devices. Besides of the tunnelling barrier, spin polarizer, and crystal quality of GaN are also crucial for the spin injection and resulted in spin polarization. In the reported FM/oxides/GaN systems, the FM with larger saturation magnetization and smaller coercivity showed the higher injection efficiency [32], and the GaN with lower defect level was beneficial for the spin transport [11, 33]. For achieving the high efficiency of spin injection in GaN with h-BN tunnelling barrier, the synergy among spin polarizer, h-BN tunnelling layer, carrier concentration and crystal quality of GaN should be investigated in a systematic manner.

In this paper, spin injection and transport in n-GaN at RT were studied by using FM/h-BN spin injector. The spin relaxation time and diffusion length with various ferromagnetic electrodes were compared through the three-terminal Hanle method. Based on the four-terminal non-local spin valves, the thicknesses of h-BN and the doping concentration as well as crystal quality of GaN were optimized. The physical mechanism for their influences on the spin injection and spin transport was revealed.

## Materials and Methods

H-BN thin films were grown on Cu foil as substrate and catalyst at 1050 °C in a low-pressure chemical vapour deposition system, and ammonia borane was used as the precursor. The as-grown h-BN film was transferred onto GaN surface through a typical wet transfer method assisted by poly (methyl methacrylate) (PMMA). A vacuum annealing at 400 °C was then carried out for half an hour to remove the residue. Electrode patterns of the three-terminal devices were constructed on GaN surface using the maskless laser direct writing technique, and the channel width was designed to be 50 μm, far beyond the spin diffusion length in GaN. The four-terminal spin valve devices were fabricated by using the electron beam lithography (EBL). The central two ferromagnetic electrodes of the four-terminal devices were, respectively, 500 nm and 800 nm in width and separated by a 600 nm channel. The two reference electrodes were 2 μm in width and 7 μm apart from the neighbouring ferromagnetic electrodes, respectively. Fe, Co, and CoFeB films with a thickness of about 20 nm were, respectively, deposited on GaN surfaces through the magnetron sputtering. The ferromagnetic electrodes were covered by a 50 nm Ru capping layer to prevent oxidation.

Optical morphology of the devices was observed on a Motic BA310Met-T microscope. Surface morphology of the h-BN on GaN was obtained using a scanning electron microscope (SEM, Carl Zeiss, Sigma-HD) and a atomic force microscope (AFM, Cypher S, Asylum Research). Raman spectrum was characterized on a Horiba LabRam HR Evolution confocal spectrometer with a 532 nm excitation laser combined with a × 100 objective confocal spectrometer. Crystalline characterization was performed on a field emission TEM (JEM-2100) operating at an accelerating voltage of 200 kV. Hysteresis loops of the FM films were detected on a vibrating sample magnetometer (LakeShore-7404). The current–voltage (I-V) curves were measured on a direct current (DC) power supply (Agilent B2901A). All the experiments were performed in air and at RT.

## Results and Discussion

**Fig. 1:**
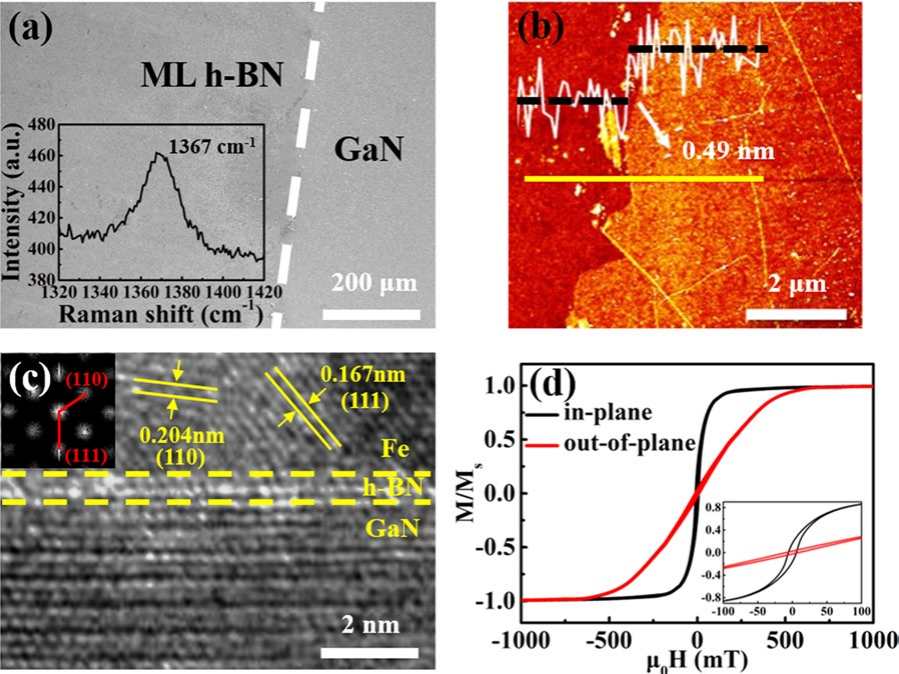
(**a**) Optical micrograph of monolayer h-BN (denoted as ML h-BN in the figure) transferred on GaN (Inset: Raman spectrum of monolayer h-BN grown on copper foil); (**b**) AFM image and corresponding height profile of monolayer h-BN; (**c**) Cross-sectional TEM image of the Fe/h-BN/GaN spin injection structure (Inset: FFT image of the Fe region); (**d**) In-plane and out-of-plane hysteresis loops of the Fe film grown on h-BN/GaN (Inset: partial enlarged hysteresis loops near the zero point)

H-BN films grown by chemical vapour deposition (see Additional file 1: Fig. S1) were transferred onto the Ga-face GaN as the tunnelling layers. The morphology and structural properties closely associated with the spin injection were characterized. As shown in Fig. 1a, b, the optical micrograph, as well as the AFM image, illustrates a smooth surface of the h-BN tunnelling layer. From the height profile in the inset of Fig. 1b, the thickness is measured as ~ 0.49 nm, corresponding to a monolayer h-BN. Raman spectrum in the illustration of Fig. 1a exhibits a characteristic vibration peak at 1367 cm^−1^, suggesting a high crystal quality of the h-BN film. Owing to the lack of dangling bond, h-BN provided a clean and stress-free surface for the subsequent growth of spin injection material avoiding the problem of lattice mismatch. For instance, the Fe film displays a nice crystal equality when growing on h-BN/GaN surface, as demonstrated by the cross-sectional TEM image in Fig. 1c. The darker areas in the top and bottom represent the Fe and GaN layers, respectively, which are separated by a brighter BN layer in the middle. Atomically sharp interfaces of the three layers without visible interdiffusion can be clearly identified. The lattice fringes of the Fe film could be recognized from the high-resolution TEM. The interplanar spacings of the crystals are 0.204 nm and 0.167 nm, respectively, corresponding to the (110) and (111) planes of the body-centred cubic α-Fe lattice, and these crystal planes could also be distinguished from the fast Fourier transform (FFT) image in the insert and the inverse fast Fourier transform (IFFT) image in Additional file 1: Fig. S2. Figure 1d depicts the hysteresis loops of the Fe film, where the in-plane and out-of-plane saturation magnetizations M_s_ are comparable, and the squareness ratio (M_r_/M_s_) is 0.16 and 0.02, respectively. The in-plane squareness is an order of magnitude higher than that of the out-of-plane, which indicates an in-plane magnetic anisotropy of the Fe film. The smooth and symmetric curves suggest a continuous rotation of the magnetic moment without pinning effect induced by the coarse domains. This also demonstrates a good crystal quality and magnetic property of the Fe film grown on h-BN.

**Fig. 2:**
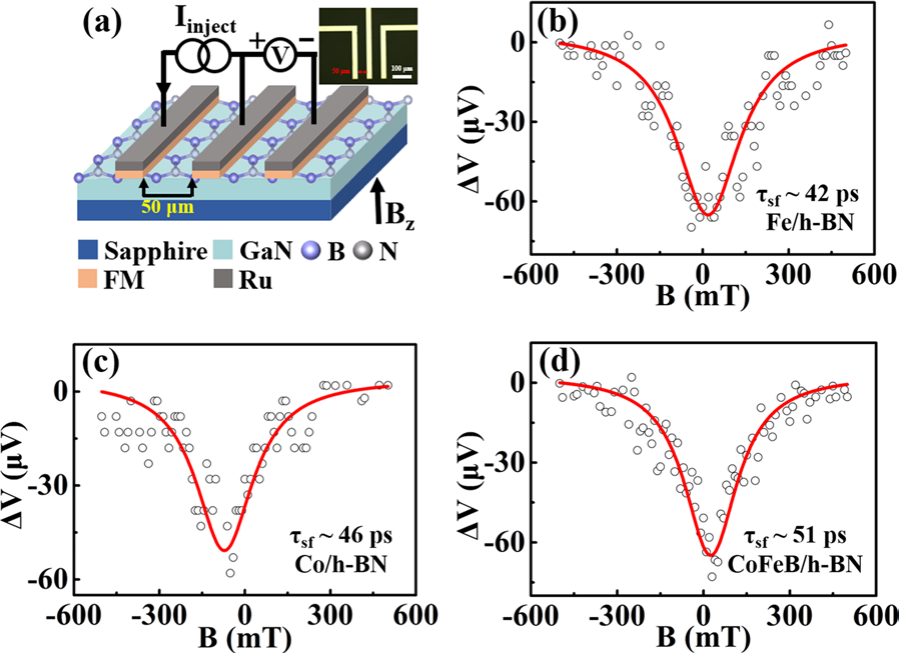
(**a**) Schematic and optical image (inset) of the three-terminal spin valve devices; (**b**, **c**, **d**) Hanle curves of the Fe/h-BN/GaN, Co/h-BN/GaN, and CoFeB/h-BN/GaN three-terminal spin valve devices at RT, respectively

As demonstrated above, h-BN can form a superior sharp and clean interface with both GaN and FM material, which is to the benefit of high-efficient spin injection and transport. Based on the h-BN tunning layer, the three-terminal spin valves were constructed on n-GaN (carrier concentration of 10^18^ cm^−3^) to investigate the spin relaxation time and spin diffusion length, as shown in Fig. 2a. During the measurement, a magnetic field is applied in the perpendicular direction of the spin polarization plane. The spin precession at the Larmor frequency will cause spin dephasing, thus making the electrochemical potential difference Δμ decrease to zero with the external magnetic field B_z_. By fitting the Hanle curve with Eqs. (1) and (2), the spin relaxation time τ_sf_ and spin diffusion length λ_sf_ can be obtained [34, 35].

1 $$\Delta \mu \left( {B_{z} } \right) = \frac{{\Delta \mu \left( 0 \right)}}{{(1 + \left( {\omega _{1} {\tau_{sf}} } \right)^{2} )}}$$

2 $${\lambda }_{sf}={(D{\tau }_{sf})}^{1/2}$$

where Δ*μ* (0) is the electrochemical potential difference under zero magnetic field, *ω*_l_ = *gμ*_*B*_*B*_*z*_/*ħ* is the spin precession frequency (*g* = 1.94 is the Landé factor of GaN) [12], and *D* is the diffusion constant.

The spin injection is strongly dependent on the ferromagnetic electrodes. To clarify this influence, about 20 nm Fe, Co, and CoFeB injection electrodes with a Ru capping layer were, respectively, fabricated on GaN surface. By detecting the Hanle precession at RT, the performance of devices with the three different ferromagnetic electrodes is compared. Since the spin injection can be current and voltage dependent in a larger bias current, which is especially pronounced for h-BN tunnel barriers[36]. To eliminate this influence, a fixed and relatively smaller bias current of 10 μA is used during the measurement. As shown in Fig. 2b, c, the three-terminal device with Fe (Co) ferromagnetic electrode produces a 65 (52) μV spin accumulation signal. The calculated spin relaxation time and spin diffusion length are 42 (46) ps and 165 (173) nm, respectively, which are significantly increased than the cases of using the Al_2_O_3_ tunnelling layer [5, 6]. This is due to the smooth two-dimensional nature of the h-BN film and the sharp van der Waals interface for the Fe (Co)/h-BN/GaN trilayer. With respect to both Fe and Co, the device using CoFeB as a ferromagnetic electrode achieves the expected longest spin relaxation time of 51 ps and the largest spin diffusion length of 182 nm, as shown in Fig. 2d, which can be attributed to its high intrinsic spin polarization and better magnetic property (see Additional file 1: Fig. S3) [13].

**Fig. 3:**
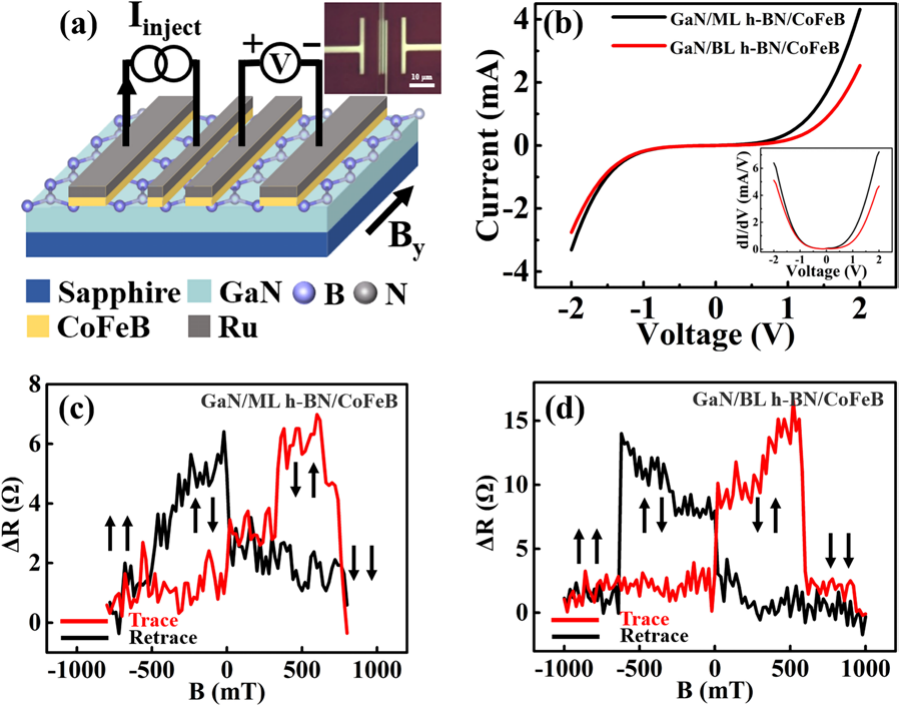
(**a**) Schematic and optical image (inset) of the four-terminal spin valve devices; (**b**) Two-terminal I–V test at RT, where the black and red lines represent the devices with monolayer and bilayer h-BN (denoted as BL h-BN in the figure) as the tunnelling barriers, respectively; (**c**, **d**) ΔR as a function of in-plane magnetic field for the devices with monolayer and bilayer h-BN as the tunnelling barriers at RT, respectively

Given the superior performance of the three-terminal device with CoFeB ferromagnetic electrode, the GaN-based four-terminal non-local spin valves with CoFeB/h-BN spin injector were further studied to reveal the spin injection efficiency. Furthermore, h-BN has been shown to be a preferred tunning layer, and its thickness was also optimized. The devices were constructed through the EBL followed by a magnetron sputtering of 20/50 nm CoFeB/Ru layer, as shown in Fig. 3a. Figure 3b displays the I-V curves detected on the central two terminals of the devices, and the results using monolayer and bilayer h-BN tunning barrier are compared. Both the I-V curves display clearly nonlinear properties, indicating the presence of a Schottky barrier at the tunnelling interface. The inset depicts the corresponding differential conductivities, where the parabolic shape is typical of tunnel junctions. It is worth noting that the device with a bilayer tunnelling layer shows a lower slope, indicative of a larger tunning barrier and a higher resistance than that with monolayer h-BN. During the magnetoresistance measurement, by applying a constant bias current to the left ferromagnetic electrode and reference electrode, the spin-polarized electrons are injected through h-BN into GaN. Since the middle injection and detection electrodes are designed with different aspect ratios (Fig. 3a), the magnetization orientation of the middle two electrons will be tuned between parallel and antiparallel when an in-plane magnetic field sweeps from + y to − y direction. Accordingly, the magnetic field-dependent voltage signals can be measured. To eliminate the influence from the background resistances, a high-precision constant current source and a lock-in amplifier are employed to collect the data during the measurement of magnetic resistance, to avoid the influence of spurious effects and make sure the accuracy of the results. With a 10 μA injected current (I_inject_), the measured voltage changes (ΔV) under various in-plane magnetic fields are provided in Additional file 1: Fig. S4a, b, and the deduced non-local magnetic resistance signals (Δ*R* = Δ*V*/*I*_inject_) are shown in Fig. 3c, d for the two devices, respectively. In the case of monolayer h-BN tunnelling barrier, the peak ΔR is about 5 Ω, while the device with bilayer h-BN exhibits a higher peak ΔR of 13 Ω, indicating a superior spin injection and transport performance of the latter.

The spin-dependent magnetoresistance and spin polarization *P*_J_ comply the following relationship [11]:

3 $$\mathrm{\Delta R}= {P}_{J}^{2} \rho \frac{{\lambda }_{N}}{A}{\mathrm{ e}}^{-L/{\lambda }_{N}}$$

where *ρ* is the resistivity of GaN, *λ*_*N*_ is the spin diffusion length, *A* is the cross-sectional area of the transport current in GaN, and *L* = 600 nm is the distance between the spin injector and detector. Since the GaN thickness is far larger than the carrier diffusion length, we perform a theoretical simulation to reveal the effective cross-sectional area of the transport current (see Additional file 1: Fig. S5). By employing the above parameters (a effective cross-sectional area of 1.2 × 10^–12^ m^2^, a resistivity of 0.11 Ω·m for ~ 10^18^ cm^−3^ n-GaN measured through the four-probe method), the spin polarization is estimated to be about 9.0% and 14.5% for the two devices, respectively. To our knowledge, the spin polarization value with bilayer h-BN is higher than any previous report for the spin injection in GaN [5, 11–13]. There are two possible reasons for the better performance of bilayer h-BN. Firstly, the bilayer h-BN has a higher tunnelling potential barrier compared with the monolayer, which can well overcome the conductivity mismatch between the ferromagnetic electrode and semiconductor. Secondly, owing to the weak van der Waals interaction, the bilayer can better alleviate the roughness of the original GaN, providing a smoother surface for the subsequent growth of the ferromagnetic electrode. As a result, spin depolarization caused by the magnetostatic fringe field of the ferromagnetic electrode can be further reduced [23]. If further increased the thickness of h-BN layer, unavoidable breakage and contamination can be produced during the transfer process, which could induce more spin scattering in the interface and even suppress the spin polarization instead.

**Fig. 4:**
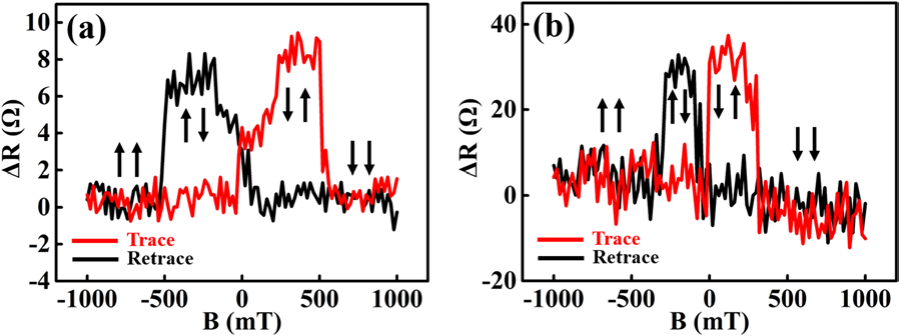
(**a**) ΔR dependence on the in-plane magnetic field for the grown n-GaN with a carrier concentration of ~ 10^17^ cm^−3^; (**b**) ΔR dependence on the in-plane magnetic field for the free-standing single-crystal n-GaN with a carrier concentration of ~ 10^18^ cm^−3^

In addition to the significant role played by ferromagnetic electrodes and tunnelling barrier, the carrier concentration of GaN is critical to the spin transport. In this consideration, n-GaN with lower carrier concentrations was employed as the substrate, to compare the spin polarization with that of the ~ 10^18^ cm^−3^ n-GaN. Based on the four-terminal non-local spin valve device, the non-local ΔR as a function of in-plane magnetic field for CoFeB/ML h-BN/GaN (~ 10^17^ cm^−3^) is shown in Fig. 4a (the as-measured magnetic resistance curve is shown in Additional file 1: Fig. S4c), where the peak ΔR is about 9 Ω. By introducing the related parameters for the ~ 10^17^ cm^−3^ n-GaN, including the four-probe measured resistivity of 0.57 Ω·m, the effective cross-sectional area of 2 × 10^–12^ m^2^ (see Additional file 1: Fig. S5), and the spin diffusion length of 195 nm (derived from the Hanle measurement), the yielding spin polarization is about 5.9%. This value is 3.1% lower than the aforementioned ~ 10^18^ cm^−3^ n-GaN with monolayer h-BN tunnelling barrier. If the carrier concentration is further reduced, the spin transport and the polarization are intensively suppressed instead, leading to an undetectable signal (not shown). Normally, a lower carrier concentration has a weaker spin relaxation caused by the doping atoms, which should be beneficial to a higher spin polarization. However, the decreased carrier concentration can also reduce the conductive channels accessible for the spin transport, resulting in a lower spin polarization instead. Figure 4b displays the ΔR signal for the spin injection in free-standing single-crystal n-GaN (~ 10^18^ cm^−3^), with the measured ΔV provided in Additional file 1: Fig. S4d. The peak ΔR is increased to be as large as 37 Ω, which is much larger than that in GaN film grown on sapphire surface. This is attributed to the high quality of the free-standing GaN crystal, in which the defect-induced spin scattering is significantly restrained during the spin transport.

**Fig. 5:**
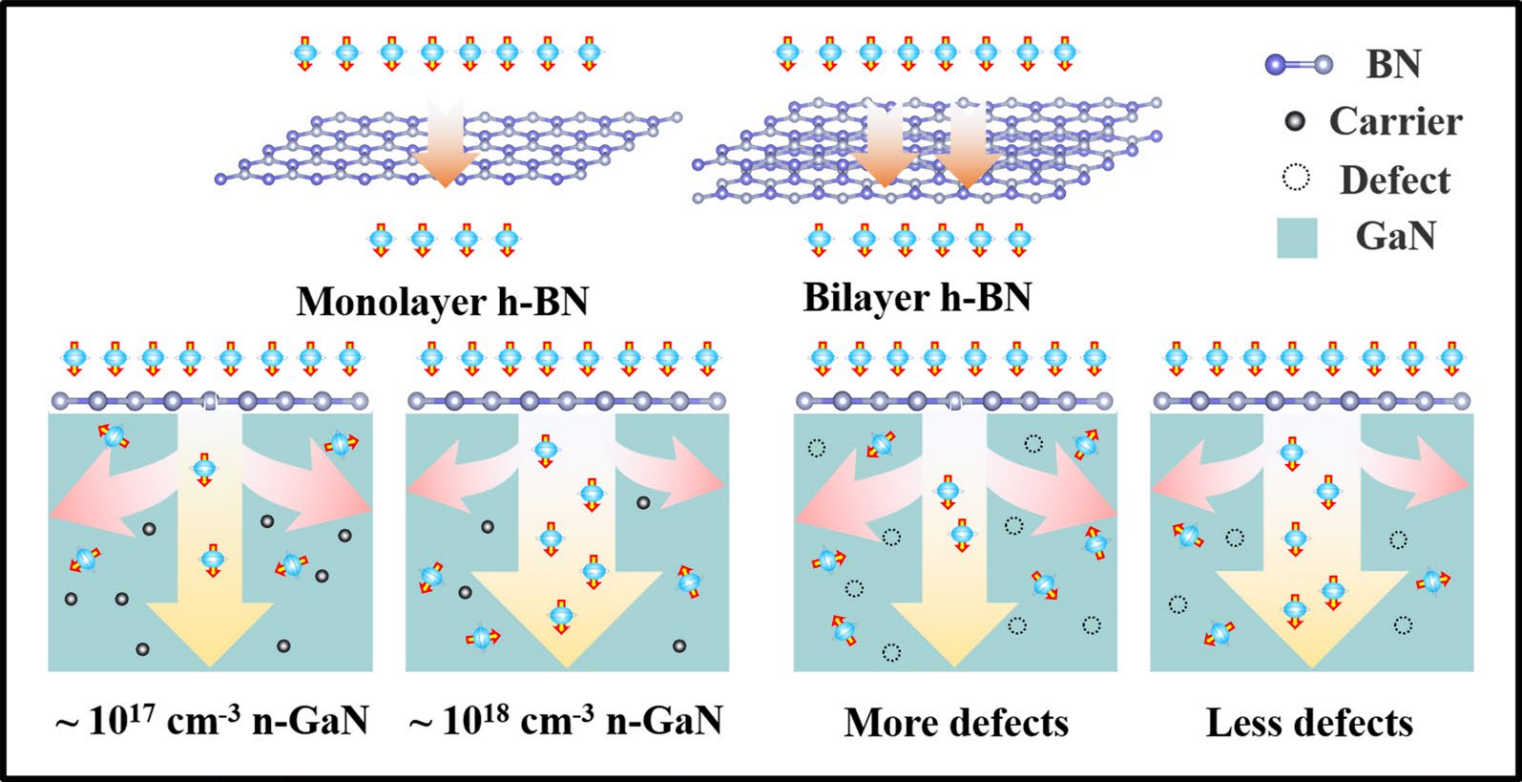
Schematic showing the effects of h-BN layer number, carrier concentration and crystal equality of GaN on the spin injection and transport

Figure 5a summarizes the mechanism of the spin injection and transport dependence on the h-BN tunnelling barrier, and the carrier concentration as well as crystal equality of GaN. The systematic understanding and the maximal spin polarizations obtained at RT in MOCVD GaN film could be considered as a critical move for the practical applications of semiconductor spintronics.

## Conclusions

In summary, spin injection in n-GaN was systematically studied with FM/h-BN spin injector at RT. By using CoFeB as the spin polarizer, the longer spin relaxation time and diffusion length were observed compared with that of Fe and Co spin polarizer. Based on the non-local spin valves measurements, CoFeB/h-BN/GaN with bilayer h-BN tunnelling barrier displayed a much higher spin polarization than the case of monolayer. Moreover, appropriate carrier concentration (~ 10^18^ cm^−3^), and better crystal quality of n-GaN could dramatically reduce the defect-induced spin scattering, resulting in a large improvement of the spin transport and spin polarization. Therefore, a record high spin polarization was achieved in the optimal CoFeB/h-BN/GaN structure at RT. All these results will open up broad prospects for the development of nitride-based spintronic devices.

## Supplementary Information

**Additional file 1**: Supplementary Information for SEM image of h-BN, IFFT image of the Fe region for the TEM image, hysteresis loops of the CoFeB film, the measured voltage signals for the spin valve devices and simulation of injected current distribution along the z direction of GaN film.

## Data Availability

The datasets used and/or analysed during the current study are available from the corresponding author on reasonable request.

## Abbreviations

H-BN: Hexagonal boron nitride
GaN: Gallium nitride
CoFeB: Cobalt ferrum boron
FM: Ferromagnetic metals
Al_2_O_3_: Aluminium oxide
MgO: Magnesium oxide
RT: Room temperature
PMMA: Polymethyl methacrylate
EBL: Electron beam lithography
SEM: Scanning electron microscope
AFM: Atomic force microscope
TEM: Transmission electron microscope
I–V: Current–voltage
DC: Direct current
*Ms*: Saturation magnetizations
Δ*μ*: Electrochemical potential difference
*B*_*z*_: External magnetic field
*τ*_*sf*_: Spin relaxation time
*λ*_*sf*_: Spin diffusion length
Δ*μ* (0): Electrochemical potential difference under zero magnetic field
*ω*_l_: Spin precession frequency
*g*: Landé factor of GaN
*μ*_*B*_: Bohr magneton
*ħ*: Reduced Planck constant
*D*: Diffusion constant
*P*_*J*_: Spin polarization
ΔR: Spin-dependent resistance signal
ΔV: Voltage change
*I*_inject_: Spin injection current
*ρ*: The resistivity of GaN
*λ*_*N*_: Spin diffusion length
*A*: The cross-sectional area of the GaN
*L*: The distance between the spin injector and detector
ML: Monolayer
BL: Bilayer
MOCVD: Metal-organic chemical vapour deposition
