# Supplementary material for: High-Efficient Spin Injection in GaN at Room Temperature Through A Van der Waals Tunnelling Barrier
Source: Nanoscale Res Lett. 2022 Aug 15;17:74. doi: 10.1186/s11671-022-03712-5 (PMC9378808; doi:10.1186/s11671-022-03712-5)
Supplement: Supplementary file 1 — Additional file 1: Supplementary Information for SEM image of h-BN, IFFT image of the Fe region for the TEM image, hysteresis loops of the CoFeB film, the measured voltage signals for the spin valve devices and simulation of injected current distribution along the z direction of GaN film. [file 11671_2022_3712_MOESM1_ESM.docx]

**Supplementary Information**

**High-efficient spin injection in GaN at room temperature through a van der Waals tunnelling barrier**

Figure S1 shows the scanning electron microscope (SEM) image of an as-grown h-BN on copper foil. The globally uniform contract indicates that the h-BN achieves a wide range of full-coverage growth.


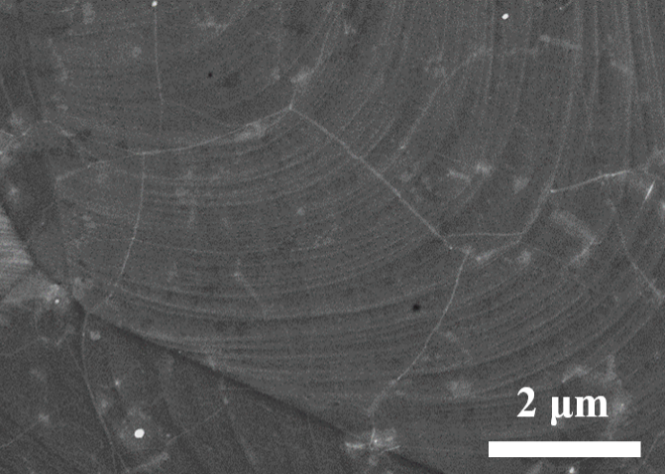


**Figure S1 SEM image of h-BN grown on copper foil.**

Figure S2 shows the inverse fast Fourier transform (IFFT) in the Fe region for the TEM image to identify the lattice configuration more clearly.


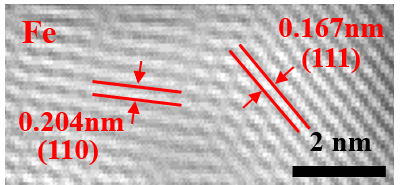


**Figure S2 IFFT image of the Fe region for the TEM image.**

Figure S3 depicts the hysteresis loops of CoFeB film grown on h-BN/GaN, where the squareness ratios (M_r_/M_s_) for the in-plane and the out-of-plane are 0.4 and 0.12, respectively, showing an in-plane magnetic anisotropy. Compared with that of the Fe film on h-BN/GaN, the squareness ratios in the two directions of CoFeB are both increased, and the in-plane magnetic anisotropy is enhanced as well, which indicate a superior magnetic property of the CoFeB film.


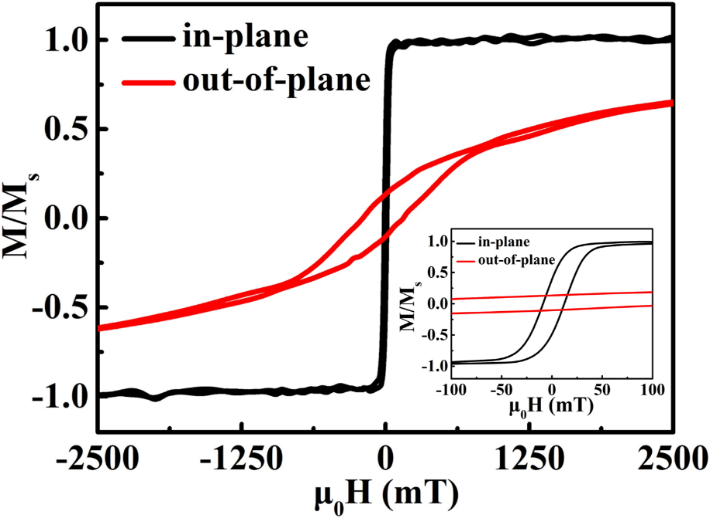


**Figure S3 In-plane and out-of-plane hysteresis loops of the CoFeB film grown on h-BN/GaN (Inset: partial enlarged hysteresis loops near the zero point).**

Figure S4 shows the measured voltage signals as a function of in-plane magnetic field for the four-terminal non-local spin valve devices. During the measurements, a bias current of 10 μA is applied.
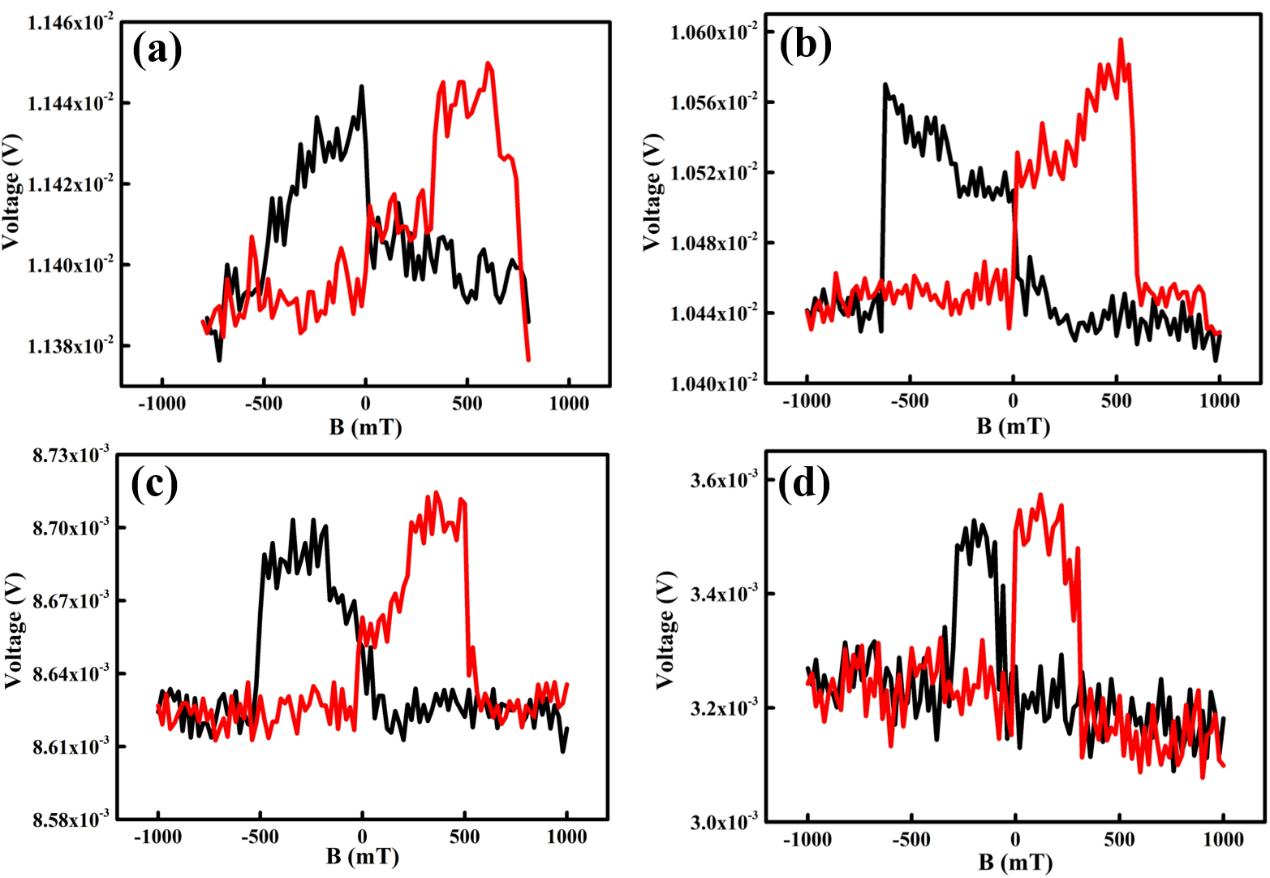


**Figure S4 (a, b) The as-measured RT magnetic resistance (MR) curves for devices based on ~ 10^18^ cm^-3^ n-GaN film, with monolayer and bilayer h-BN as the tunneling barriers, respectively; (c) The as-measured RT MR curve for device based on ~ 10^17^ cm^-3^ n-GaN film, with monolayer h-BN as the tunneling barrier at RT; (d) The as-measured RT MR curve for device based on free-standing single-crystal n-GaN, with monolayer h-BN as the tunneling barrier at RT.**

To estimate the effective cross-sectional area of the current transport, a commercial Technology Computer Aided Design (TCAD) software (SILVACO ATLAS) is employed to simulate the distribution of injected current along the z direction of the GaN film. The structure models are constructed using the practical geometric and electrical parameters in the experiments, and the simulated results are shown in Figure S5 (a, b) for the cases of ~ 10^17^ cm^-3^ and ~ 10^18^ cm^-3^ n-GaN, respectively. As can be seen for both cases, most of the current density distributes in a thin region near the GaN surface, especially at around the electrode edges. When going deep into the GaN film, the current density decreases significantly. At a depth of 0.1 µm for ~ 10^17^ cm^-3^ n-GaN film and 0.06 µm for ~ 10^18^ cm^-3^ n-GaN film, the current attenuation exceeds more than one order of magnitude. Accordingly, the effective channel depth for the transport current in the GaN films is estimated to be about 0.1 µm for ~ 10^17^ cm^-3^ n-GaN film and about 0.06 µm for ~ 10^18^ cm^-3^ n-GaN film, respectively. The effective cross-sectional area of the transport current is calculated to be 2×10^-12^ m^2^ and 1.2×10^-12^ m^2^, for ~ 10^17^ cm^-3^ and ~ 10^18^ cm^-3^ n-GaN films, respectively, by multiplying the effective channel depth by channel length.


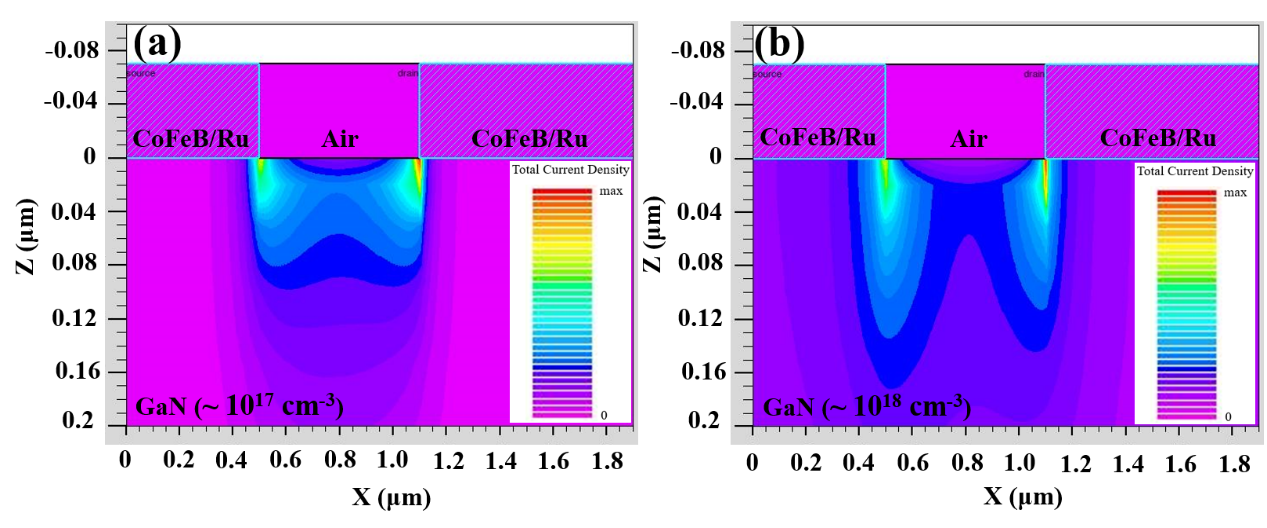


**Figure S5 (a, b) Distribution of injected current along the z direction of the ~ 10^17^ cm^-3^ and ~ 10^18^ cm^-3^ GaN films, respectively.**
